# Cancer aggravation due to persistent pain signals with the increased expression of pain-related mediators in sensory neurons of tumor-bearing mice

**DOI:** 10.1186/s13041-023-01001-5

**Published:** 2023-02-03

**Authors:** Kenichi Tanaka, Takashige Kondo, Michiko Narita, Takeru Muta, Sara Yoshida, Daisuke Sato, Yukari Suda, Yusuke Hamada, Takatsune Shimizu, Naoko Kuzumaki, Minoru Narita

**Affiliations:** 1 Present Address: Department of Pharmacology, Hoshi University School of Pharmacy and Pharmaceutical Sciences, 2-4-41 Ebara, Shinagawa-ku, Tokyo, 142-8501 Japan; 2 Division of Cancer Pathophysiology, National Cancer Center Research Institute, 5-1-1 Tsukiji, Chuo-ku, Tokyo, 104-0045 Japan; 3 Department of Pathophysiology, Hoshi University School of Pharmacy and Pharmaceutical Sciences, 2-4-41 Ebara, Shinagawa-ku, Tokyo, 142-8501 Japan

**Keywords:** Cancer pain, Sensory neuron, Tumor growth, Survival, Cancer-neuron interaction

## Abstract

A growing body of evidence suggests that intractable pain reduces both the quality of life and survival in cancer patients. In the present study, we evaluated whether chronic pain stimuli could directly affect cancer pathology using tumor-bearing mice. For this purpose, we used two different models of chronic pain in mice, neuropathic pain and persistent postsurgical pain, with Lewis lung carcinoma (LLC) as tumor cells. We found that tumor growth was dramatically promoted in these pain models. As well as these pain models, tumor growth of LLC, severe osteosarcoma (AXT) and B16 melanoma cells was significantly promoted by concomitant activation of sensory neurons in AAV6-hM3Dq-injected mice treated with the designer drug clozapine-*N*-oxide (CNO). Significant increases in mRNA levels of vascular endothelial growth factor-A (Vegfa), tachykinin precursor 1 (Tac1) and calcitonin-related polypeptide alpha (Calca) in the ipsilateral side of dorsal root ganglion of AAV6-hM3Dq-injected mice were observed by concomitant activation of sensory neurons due to CNO administration. Moreover, in a model of bone cancer pain in which mice were implanted with AXT cells into the right femoral bone marrow cavity, the survival period was significantly prolonged by repeated inhibition of sensory neurons of AAV6-hM4Di-injected mice by CNO administration. These findings suggest that persistent pain signals may promote tumor growth by the increased expression of sensory-located peptides and growth factors, and controlling cancer pain may prolong cancer survival.

**Supplementary Information:**

The online version contains supplementary material available at 10.1186/s13041-023-01001-5.

## Introduction

Cancer pain is caused by pressure from the tumor on bones, nerves, or other organs in the body [1]. Cancer treatment, such as surgery, radiation therapy or chemotherapy, may also produce pain [1, 2]. In late-stage cancer, bone metastases often produce even more severe pain [3, 4]. Cancer pain may be difficult to manage because it can be caused by a variety of factors. It has been reported that more than half of cancer patients experience pain, and in the late stage most cancer patients experience moderate to severe pain [5, 6]. Recent studies have shown that persistent pain reduces patients’ quality of life (QOL) and decreases survival rates in a variety of diseases [7–11]. These reports suggest that cancer pain contributes to worsening of the cancer pathology. Furthermore, among patients with metastatic non-small-cell lung cancer, early palliative care can lead to significant improvements in QOL [12]. However, while pain relief is a critical component of care for cancer patients, pain is often not easily relieved by currently available drug therapies.

It has recently been documented that peripheral nerves extend into the tumor tissue and play a role in the control of cancer progression [13, 14]. These sensory neurons are also believed to partly correspond to tumor progression by interacting with various cells that constitute the tumor microenvironment [15].

In the present study, we investigated whether persistent pain stimuli could aggravate cancer pathology in tumor-bearing mice. Additionally, we also evaluated whether concomitant activation of sensory neurons by chemogenetic manipulation could lead to changes in sensory neurons of tumor-bearing mice. Finally, we demonstrated whether the repeated inhibition of sensory neurons could improve the survival period in a model of bone cancer pain.

## Materials and methods

### Animals

Male C57BL/6J mice (6–23 weeks old) (Tokyo Laboratory Animals Science Co., Ltd., Tokyo, Japan) used in the present study were allowed access to food and water ad libitum and maintained on a 12 h:12 h light/dark cycle (light on at 8 a.m.) in a temperature- and humidity-controlled room (24 ± 1 °C, 55 ± 5%). All mice were housed in groups of 2–6 mice. All experiments were conducted in accordance with the Guide for the Care and Use of Laboratory Animals of Hoshi University School of Pharmacy and Pharmaceutical Sciences, which is accredited by the Ministry of Education, Culture, Sports and Technology of Japan.

### Production of a model of neuropathic pain by sciatic nerve ligation

Under 3% isoflurane anesthesia (FUJIFILM Wako Pure Chemical Co. LTD, Osaka, Japan), tight ligation of the sciatic nerve in the right hind limb of mice was performed by using 8-0 silk suture around approximately one-half of the diameter of the nerve, as previously described [16, 17]. In sham-operated mice, the sciatic nerve was just exposed but not ligated. To prevent dehydration after surgery, water and food were available ad libitum.

### Creation of a model of persistent postsurgical pain by electrocautery

Under isoflurane anesthesia, a 3-mm longitudinal incision was made in the skin and fascia of the right hind paw of a mouse using a No. 23 scalpel blade from 3 mm from the proximal end of the heel to the toe. Next, with the use of a monopolar electrosurgery unit (at 50 W; Vetroson^®^ V-10; Summit Hill Laboratories, NJ, USA) with a dispersive electrode pad placed under the mouse's body, a 3-mm longitudinal incision was made on the plantaris muscle, as previously described [18]. Electrocautery was performed while maintaining coagulation and hemostasis of the incision during dissection. The skin was stitched with two mattress sutures of 7-0 nylon. In sham operated mice, the plantaris muscle was exposed without the incision, and the skin was stitched with a simple interrupted suture of 7-0 nylon.

### Chemogenetic manipulation of sensory neurons

Under isoflurane anesthesia, a 1–2 cm longitudinal incision was made in the skin and the connective tissue between the gluteus superficialis and biceps femoris muscles to expose the sciatic nerve in the right hind limb (ipsilateral side) of a mouse, as previously described [19]. Next, an internal cannula (Eicom Co., Kyoto, Japan) was inserted into the sciatic nerve and adeno-associated virus (AAV) vector was microinjected at 1 µL/min for 4 min (4 µL total volume) with a glass micropipette and an air pressure injector system (Micro-syringe Pump-Model ESP-32; Eicom Co.). After the surgical procedures, mice were intraperitoneally injected with clozapine *N*-oxide (CNO) (3 mg/kg, *t.i.d*., abcam, Cambridge, UK) for 2 weeks. The following viruses were used in this study: AAV6-hSyn-hM3Dq-mCherry, AAV6-hSyn-hM4Di-mCherry, AAV6-hSyn-EGFP and AAV6-hSyn-mCherry. AAV6-hSyn-EGFP and AAV6-hSyn-mCherry were used as a control vector. All of the viruses used in this study were kindly provided by Dr. Akihiro Yamanaka (Nagoya University, Nagoya, Japan).

### Immunohistochemistry

Immunohistochemistry was performed according to a previously reported protocol [17]. Over 2 weeks after AAV injection, mice were anesthetized with 3% isoflurane and intracardially perfused with 4% paraformaldehyde in 0.1 M PBS (pH 7.4; PFA). After perfusion, the lumbar spinal cord and the lumbar dorsal root ganglion (DRG) were quickly removed and post-fixed with PFA and cryoprotected in 20–30 (w/v) % sucrose (FUJIFILM Wako). The tissue sections were embedded in an O.C.T. compound (Sakura Finetec USA. Inc., CA, USA), and the sections were cut on a cryostat (15 μm for spinal cord, 8–10 μm for DRG) (CM1860; Leica Microsystems, Heidelberg, Germany). The slices were blocked in 3% normal goat serum (NGS: Vector laboratories, Inc., CA, USA) or normal horse serum (Vector laboratories)/0.1–0.2% Triton X-100 in 0.01 M PBS or 5% NGS for 1 h at RT. Then incubated with the primary antibodies used were as follows: rabbit anti-mCherry (1:1000, abcam), chicken anti-mCherry (1:2500, abcam), sheep anti-CGRP (1:1000, Enzo Life Sciences, PA, USA), rabbit anti-substance P (1:1000, ImmunoStar, WI, USA), goat anti-peripherin (1:50, Santa Cruz Biotechnology, TX, USA.) for 48 h at 4 °C or 24 h at RT. Following washes, they were incubated in goat anti-rabbit Alexa 546 (1:10,000; Thermo Fisher Scientific Inc., MA, USA), goat anti-chicken Alexa 546 (1:10,000; Thermo Fisher Scientific), donkey anti-sheep Alexa 488 (1:750; Thermo Fisher Scientific), chicken anti-rabbit Alexa 488 (1:700; Thermo Fisher Scientific), donkey anti-goat Alexa 488 (1:400; Thermo Fisher Scientific) conjugated secondary antibodies for 2 h at RT. Immunofluorescence was detected by a light microscope (BX53, Olympus, Tokyo, Japan) and captured using a high-sensitivity digital CCD camera (MD-695; Molecular Devices, CA, USA). Imaging analysis was performed using Metamorph 7.8 software (Molecular Devices).

### Plantar test

The latency of the hind paw withdrawal in response to nociceptive stimulation was measured by focusing a thermal stimulus on the plantar surface of the hind paw of mice using a thermal stimulus apparatus (model 33 Analgesia Meter; IITC/Life Science Instruments, CA, USA) as described previously [20]. Before the experiments, mice were habituated for 1 h in an acrylic cylinder (15 cm height and 8 cm in diameter).

### Measurement of mechanical allodynia

Mechanical allodynia was assessed by a plantar electronic von Frey Anesthesiometer (ALMEMO 2450 Ahlborn; IITC/Life Science). Briefly, a chip attached to the device was applied vertically to the plantar surface of the hind paw of mice, and the pressure (g) until the hind paw was flicked off was recorded as the pain threshold. Before this assessment, mice were habituated for 1 h in an acrylic cylinder (15 cm height and 8 cm diameter) on an elevated mesh floor.

### Cell culture

Lewis lung carcinoma (LLC), LLC-luc and B16 melanoma (B16) cells were cultured in Minimum Essential Medium Alpha (α-MEM) (Thermo Fisher Scientific) supplemented with 10% fetal bovine serum (FBS) (Thermo Fisher Scientific) and 1% penicillin/streptomycin (Thermo Fisher Scientific). LLC-luc cells were generated by infection of LLC cells with lentiviral vector expressing ff-Luc genes. Mouse severe osteosarcoma (AXT) cells were established as previously described [21, 22]. AXT cells and AXT-luc cells were cultured under 5% CO_2_ at 37 °C in Iscove’s modified Dulbecco’s medium (IMDM) (Thermo Fisher Scientific) supplemented with 10% FBS.

### Graft tumor growth assay

LLC, LLC-luc, B16 and AXT cells were counted after trypsinization and then resuspended in a mixture of extracellular matrix gel (Sigma-Aldrich Inc., MO, USA) and Hank’s Balanced Salt Solution (HBSS) (Thermo Fisher Scientific) at a concentration of 5 × 10^5^ cells/0.15 mL as appropriate, according to a previously reported protocol [23, 24]. These suspensions in a volume of 0.15 mL were inoculated subcutaneously into the right thigh close to the sciatic nerve of mice under 3% isoflurane anesthesia. Tumor size was measured using a caliper and tumor volume was calculated as (L × W^2^)/2, where L = length and W = width.

### Cancer pain model mice

To establish tumor xenografts, AXT-luc cells (1 × 10^6^ cells) suspended in 50 μL of IMDM were injected into the right femoral bone marrow cavity of syngeneic C57BL/6J mice, as previously described [16]. Briefly, the knee joint was flexed to 90° and the distal side of the femur was exposed by incising the skin. A 23-gauge needle was inserted into the bone marrow cavity to make a small hole, into which AXT-luc cells or medium alone were injected. All procedures were performed under inhalational anesthesia with 3% isoflurane.

### In vivo imaging system (IVIS)

Approximately 10 min before imaging, the substrate luciferin was injected into the intraperitoneally at 4.5 mg/mouse (15 mg/ml, FUJIFILM Wako). Mice were anesthetized with isoflurane/oxygen and placed on the imaging stage. Dorsal images were acquired using the IVIS^®^ Lumina Series III (PerkinElmer Inc., MA, USA).

### Quantitative reverse transcription polymerase chain reaction (RT-qPCR)

RT-qPCR was performed according to our previous report [18]. For RT-qPCR analysis, total RNAs were isolated from the ipsilateral side of the mouse dorsal root ganglions (DRG) (L3–L5) and then first-strand cDNAs were synthesized. Glyceraldehyde 3-phosphate dehydrogenase (Gapdh) was used as an internal control. Additional file 1: Table S1 represents a complete list of all primers used in this study.

### Statistical analysis

The results are shown as the mean ± standard error of the mean (S.E.M.). We analyzed and described the statistical significance of differences between groups according to an unpaired *t*-test and one-way or two-way analysis of variance followed by the Bonferroni multiple comparisons test. The data were subjected to a comparative analysis by testing the null hypothesis for the Pearson product moment correlation. For survival analysis, the Log-rank (Mantel-Cox) test was used. All statistical analyses were performed with Prism version 9.0 (GraphPad software, CA, USA).

## Results

### Effect of persistent pain induced by sciatic nerve ligation or paw electrocautery operation on tumor growth

**Fig. 1:**
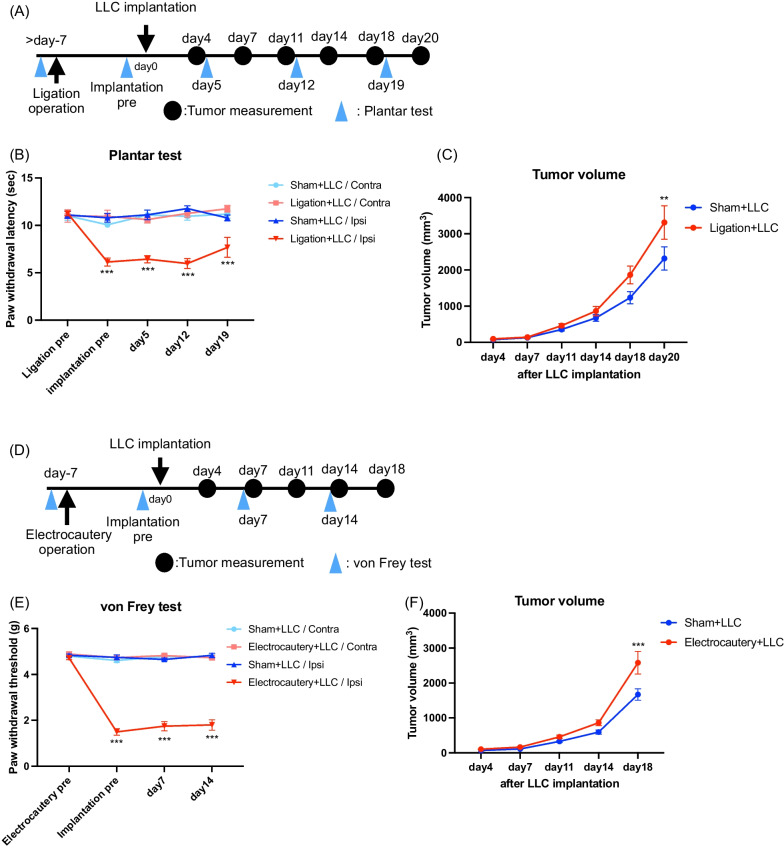
Persistent pain induced by sciatic nerve ligation or paw electrocautery operation promotes tumor growth. **A** Experimental timeline. **B** Changes in the pain threshold induced by sciatic nerve ligation under the tumor-bearing state, measured by a plantar test (n = 7–10, Two-way repeated measures ANOVA with the post-hoc Bonferroni test, ***p < 0.001 vs. Sham + LLC/Ipsi). Contra: Contralateral paw, Ipsi: Ipsilateral paw. **C** Quantitative analysis of tumor volume in the Sham + LLC and Ligation + LLC groups (n = 13–15, Two-way repeated measures ANOVA with the post-hoc Bonferroni test, **p < 0.01 vs. Sham + LLC). **D** Experimental timeline. **E** Changes in the pain threshold induced by paw electrocautery operation in the tumor-bearing state, measured by the von Frey test (n = 16, Two-way repeated measures ANOVA with the post-hoc Bonferroni test, ***p < 0.001 vs. Sham + LLC/Ipsi). **F** Quantitative analysis of tumor volume in the Sham + LLC and Electrocautery + LLC groups (n = 16, Two-way repeated measures ANOVA with the post-hoc Bonferroni test, ***p < 0.001 vs. Sham + LLC)

We first investigated the influence of activated sensory neurons as a result of sciatic nerve ligation on tumor growth (Fig. 1A). We produced partial sciatic nerve ligation as a model of neuropathic pain. Next, to create tumor-bearing mice, LLC cells were subcutaneously implanted around the sciatic nerve-ligated region at 7 days after the operation. Under these conditions, the pain threshold and tumor volume in these mice were evaluated. As a result, a significant thermal hyperalgesia response was observed in the ipsilateral side of the sciatic nerve ligation group from 7 days after surgery by a plantar test (Fig. 1B, ***p < 0.001 vs. Sham + LLC/Ipsi). Under these conditions, tumor volume was significantly increased in the sciatic nerve ligation group at 20 days after LLC implantation compared to that in sham-operated mice (Fig. 1C, **p < 0.01 vs. Sham + LLC). Next, to investigate the effects on tumor growth in different pain models, we investigated the influence of activated sensory neurons by electrocautery surgery of a hind paw as a model of persistent postsurgical pain on tumor growth (Fig. 1D). In the same experimental schedule as sciatic nerve ligation, LLC cells were implanted 7 days after surgery, and changes in pain thresholds and tumor volume were evaluated. A significant tactile allodynia was observed in the ipsilateral side of the electrocautery group from 7 days after surgery by the von Frey test (Fig. 1E, ***p < 0.001 vs. Sham + LLC/Ipsi). Under these conditions, tumor volume was significantly increased in the electrocautery group at 18 days after LLC implantation compared to that in sham-operated mice (Fig. 1F, ***p < 0.001 vs. Sham + LLC).

### Induction of transient hyperalgesia by chemogenetic manipulation of sensory nerves

**Fig. 2:**
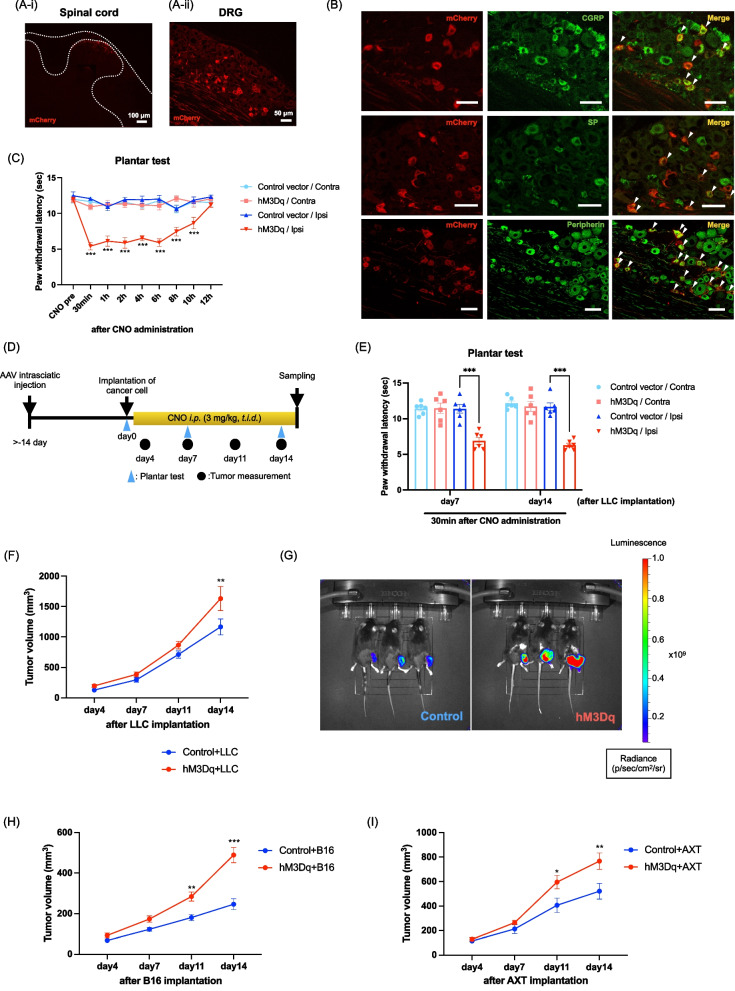
Chemogenetic manipulation of sensory nerves promotes tumor growth. **A** Qualitative observation of hM3Dq-mCherry fluorescence in histological sections. hM3Dq-mCherry (red) was expressed in the lumbar spinal cord [scale bar: 100 μm (**A-i**)] and lumbar DRG [scale bar: 50 μm (**A-ii**)]. **B** Lumbar DRG sections were stained with antibodies specific for CGRP, SP or peripherin (all shown in green). hM3Dq: red, overlay: yellow. Scale bars: 50 μm. **C** Changes in the pain threshold induced by the activation of sensory neurons of hM3Dq-expressed mice by CNO administration, measured by a plantar test (n = 6, Two-way repeated measures ANOVA with the post-hoc Bonferroni test, ***p < 0.001 vs. Control vector/Ipsi). **D** Experimental timeline. **E** Changes in the pain threshold induced by the activation of sensory neurons of hM3Dq-expressed mice by CNO administration under the tumor-bearing state, measured by a plantar test (n = 6, Two-way repeated measures ANOVA with the post-hoc Bonferroni test, ***p < 0.001 vs. Control vector/Ipsi). **F** Quantitative analysis of tumor volume in the Control + LLC and hM3Dq + LLC groups (n = 10, Two-way repeated measures ANOVA with the post-hoc Bonferroni test, **p < 0.01 vs. Control + LLC). **G** Bioluminescent images of LLC-luc tumor were used to determine the tumor size at two weeks after the implantation of LLC-luc cells. **H** Quantitative analysis of tumor volume in the Control + B16 and hM3Dq + B16 groups (n = 24–27, Two-way repeated measures ANOVA with the post-hoc Bonferroni test, **p < 0.01, ***p < 0.001 vs. Control + B16). **I** Quantitative analysis of tumor volume in the Control + AXT and hM3Dq + AXT groups (n = 10, Two-way repeated measures ANOVA with the post-hoc Bonferroni test, *p < 0.05, **p < 0.01 vs. Control + AXT)

AAV6-hSyn-hM3Dq-mCherry or control vector was injected into the sciatic nerves of mice. We confirmed the expression of hM3Dq-mCherry in the lumbar spinal cord and DRG as the projection of sensory nerves over 2 weeks after AAV injection (Fig. 2A). In addition, the expression of hM3Dq-mCherry in the DRG of AAV-injected mice was highly co-localized with markers for peptidergic C fiber neurons, calcitonin gene-related peptide (CGRP) and substance P (SP), and a marker for small unmyelinated C fiber and thinly myelinated Aδ fiber neurons, peripherin [25, 26] (Fig. 2B). Under these conditions, we found that the Gq-DREADD-mediated activation of sensory nerves by CNO administration caused thermal hyperalgesia on the ipsilateral side, but not on the contralateral side, from 30 min to 10 h after CNO administration at over 2 weeks after AAV injection (Fig. 2C, ***p < 0.001 vs. Control vector/Ipsi). On the other hand, these mice did not show spontaneous pain-like behaviors (data not shown).

### Effect on tumor growth under the chemogenetic manipulation of sensory nerves

After we confirmed that CNO administration induced pain, LLC cells were subcutaneously implanted around the sciatic nerve region over 2 weeks after AAV injection (Fig. 2D). At 7 and 14 days after LLC implantation, thermal hyperalgesia was observed at 30 min after CNO administration (Fig. 2E, ***p < 0.001 vs. Control vector/Ipsi). Under these conditions, the repeated administration of CNO significantly increased tumor volume in hM3Dq-expressing mice compared to that in control mice (Fig. 2F, **p < 0.01 vs. Control + LLC). Furthermore, to investigate the luminescence intensity in tumors, LLC-luc cells were subcutaneously implanted around the sciatic nerve of AAV-injected mice. The luminescence of luciferase in tumors was induced by intraperitoneal administration of the ligand of luciferase, luciferin. Under these conditions, the luminescence intensity was dramatically increased in hM3Dq-expressing mice compared to that in control mice at 2 weeks after tumor implantation (Fig. 2G). In other tumor-bearing models, B16 cells and AXT cells, tumor volume was significantly increased in hM3Dq-expressed mice compared to those in control mice (Fig. 2H and I, H; **p < 0.01, ***p < 0.001 vs. Control + B16, I; *p < 0.05, **p < 0.01 vs. Control + AXT).

### Influence of chemogenetic manipulation of sensory nerves on gene expression profiling of DRG neurons in tumor-bearing AAV-injected mice

**Fig. 3:**
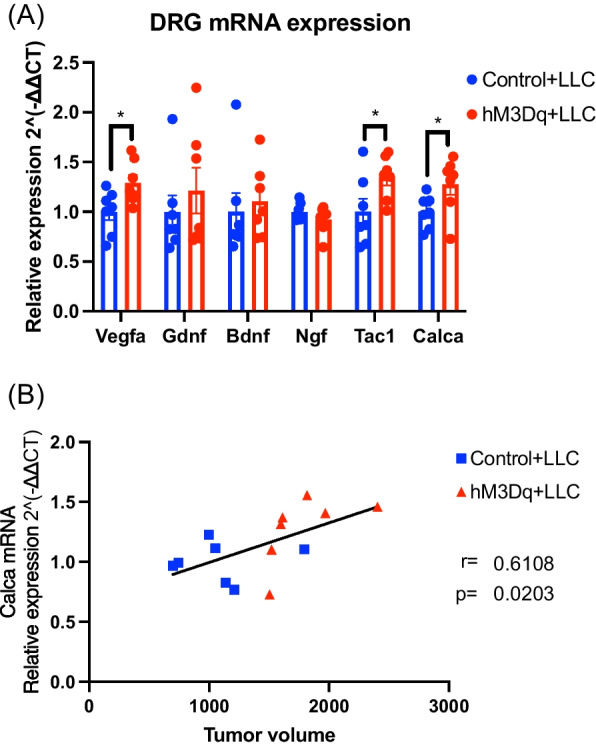
Gene expression analysis of lumbar DRG in tumor-bearing mice. **A** The mRNA levels of Vegfa, Gdnf, Bdnf, Ngf, Tac1 and Calca in the lumbar DRG at 15 days after LLC implantation were measured by RT-qPCR (n = 7, unpaired *t*-test, *p < 0.05 vs. Control + LLC). **B** Correlation between the mRNA levels of Calca in the lumbar DRG at 15 days after LLC implantation and tumor volume (r = 0.6108, p = 0.0203). The data were subjected to a comparative analysis by testing the null hypothesis for the Pearson product moment correlation. Each dot represents an individual mouse, and the line represents the regression line

The mRNA levels of vascular endothelial growth factor a (Vegfa), tachykinin precursor 1 (Tac1), encoding SP, and calcitonin-related polypeptide alpha (Calca), encoding CGRP, were significantly increased in the ipsilateral side of lumbar DRG at 15 days after LLC implantation in hM3Dq-expressing mice compared to those in control mice (Fig. 3A, *p < 0.05 vs. Control + LLC). Moreover, in the tumor-bearing condition, the mRNA level of Calca was significantly and positively correlated with tumor volume (Fig. 3B, r = 0.6108, p = 0.0203).

### Influence of the suppression of sensory neurons on survival under a cancer pain-state

**Fig. 4:**
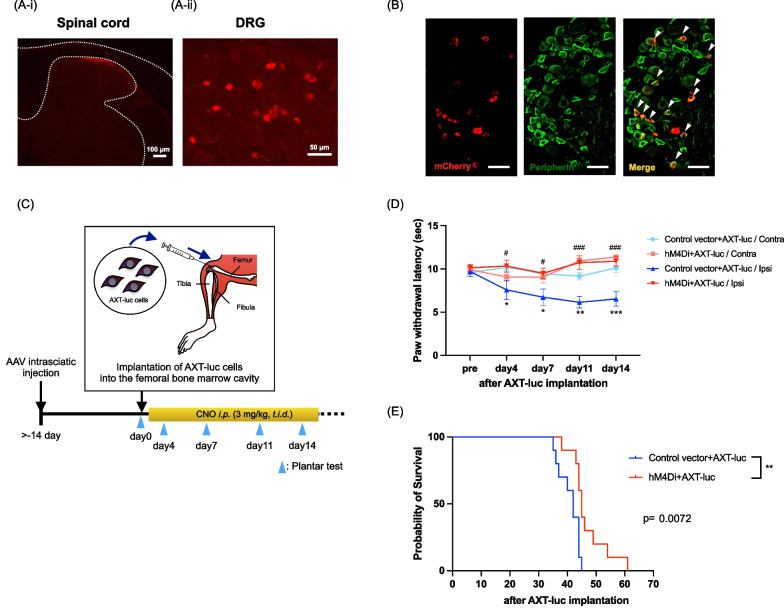
Influence of the suppression of sensory neurons on survival under a cancer pain-state. **A** Qualitative observation of hM4Di-mCherry fluorescence in histological sections. hM4Di-mCherry (red) was expressed in the lumbar spinal cord [scale bar: 100 μm (**A-i**)] and lumbar DRG [scale bar: 50 μm (**A-ii**)]. **B** Lumbar DRG sections were stained with antibodies specific for peripherin (shown in green). hM4Di: red, overlay: yellow. Scale bars: 50 μm. **C** Experimental time line and schematic illustration of AXT-luc cell implantation. **D** Changes in the cancer pain threshold suppressed by the Gi-DREADD system in AXT-luc tumor-bearing mice, measured by a plantar test (n = 9–10, Two-way repeated measures ANOVA with the post-hoc Bonferroni test, *p < 0.05, **p < 0.01, ***p < 0.001, vs. Control vector + AXT-luc/Contra, ^#^p < 0.05, ^###^p < 0.001, hM4Di + AXT-luc/Ipsi vs. Control vector + AXT-luc/Ipsi). **E** Kaplan–Meier curves showing survival of AXT-luc tumor-bearing AAV6-hM4Di- or control vector-injected mice (n = 10, Log-rank (Mantel-Cox) test, p = 0.0072)

To selectively inhibit the activity of sensory nerves, AAV-hSyn-hM4Di-mCherry or control vector was injected into the sciatic nerves of mice. We confirmed the expression of hM4Di-mCherry in the lumbar spinal cord and DRG as the projection of sensory nerves, and also confirmed the co-localization with peripherin over 2 weeks after AAV injection (Fig. 4A and B). At this point, we implanted AXT-luc cells into the intrafemoral bone marrow of AAV-injected mice to produce a model of bone cancer pain (Fig. 4C). We found that the Gi-DREADD-mediated inhibition of sensory nerves via the administration of CNO significantly inhibited thermal hyperalgesia on the ipsilateral side of AXT-luc implanted mice (Fig. 4D, *p < 0.05, **p < 0.01, ***p < 0.001, vs. Control vector + AXT-luc/Contra, ^#^p < 0.05, ^###^p < 0.001, hM4Di + AXT-luc/Ipsi vs. Control vector + AXT-luc/Ipsi). Under these conditions, the survival period of hM4Di-expressing mice was significantly prolonged by the DREADD-mediated inhibition of sensory nerves compared to that in control mice after tumor implantation (Fig. 4E, p = 0.0072).

## Discussion

It has been reported that up to 40% of cancer patients suffer from neuropathic pain [27]. This cancer-related neuropathic pain is caused by both cancer treatments such as surgery or chemotherapy and tissue destruction due to tumor growth, leading to increases in pain intensity and analgesic consumption, and to a reduced QOL [2, 27]. These reports suggest that neuropathic pain may correspond to the cancer pathology. In the present study, we first investigated whether persistent pain in a standard mouse model of neuropathic pain could affect tumor growth. We produced partial sciatic nerve ligation to induce neuropathic pain, and LLC cells were subcutaneously implanted around the sciatic nerve-ligated region at 7 days after the operation. We found that tumor volume in sciatic nerve-ligated mice was significantly increased compared to that in sham mice, suggesting that sustained activation of sensory nerves under a neuropathic pain-like state may promote tumor growth.

In general, the first choice for cancer treatment is surgery, which frequently triggers postsurgical pain [28, 29]. Moreover, it has been reported that persistent postsurgical pain due to cancer surgery results in a poor prognosis with less analgesic [30]. These findings suggest that perioperative pain management in cancer patients is important not only for preventing the persistence of postsurgical pain, but also for inhibiting cancer progression and worsening life expectancy. Therefore, we next used a model of persistent postsurgical pain induced by electrocautery [18] to further assess the influence of pain signaling on tumor growth. In the present study, the tumor volume in electrocautery-treated mice was significantly increased compared to that in sham-operated mice. Taken together, the present results suggest that, regardless of the type of pain, accelerated tumor growth can be induced in the presence of persistent pain.

With the aim of producing painful stimuli without inflammation, we attempted to establish a model that could induce nerve firing specifically in sensory nerves according to a chemogenetic approach. As a result, the activation of both peptidergic C fibers and thinly myelinated Aδ fibers by genetic manipulation with an AAV vector induced transient hyperalgesia when its specific ligand, CNO, was administered. Under these conditions, we demonstrated that the tumor volume in hM3Dq-expressing mice was dramatically increased compared to that in control mice, regardless of which cancer types, LLC, B16, or AXT cells, were used. These findings suggest that tumor enlargement in many types of tumor cells could be induced by the prolonged hyperactivity of sensory nerves.

To elucidate the mechanism of accelerated tumor growth driven by the hyperactivity of sensory neurons, we performed transcriptome profiling in DRG neurons of hM3Dq-expressing mice by RT-qPCR. We found that the mRNAs of Vegfa, Tac1 and Calca were significantly increased in hM3Dq-expressing mice by the concomitant activation of sensory nerves. VEGF in the tumor microenvironment (TME) is well known to induce angiogenesis and contributes to tumor initiation, promoting cancer progression [31–33]. On the other hand, the functions of SP and CGRP in the TME were poorly understood. However, several recent studies have reported that both SP and CGRP may directly affect cancer progression [34–36]. In particular, it has been known that CGRP may promote tumor growth via activation of extracellular signal-regulated kinases (ERKs)/Signal transducer and activator of transcription 3 (STAT3) signaling in cancer cells [37]. In fact, our result showed that there was a significant and strong positive correlation between the mRNA expression level of CGRP and tumor volume. These findings suggest that repeated activation of sensory nerves may facilitate tumor growth via an increase in the release of VEGF, SP and especially CGRP from sensory neurons at the TME.

Finally, we investigated whether the inhibition of sensory nerve activity could improve the survival period in a model of bone cancer pain. We found that the thermal hyperalgesia induced by AXT-luc implantation into the intrafemoral bone marrow was significantly inhibited by the repeated administration of CNO. Under these conditions, the survival period was prolonged by the DREADD-mediated inhibition of sensory nerves compared to that in control mice. Taken together, these findings suggest that the neural activity of sensory neurons involved in pain transmission may play a crucial role in cancer survival.

Recently, centrifugal signals from the brain have been considered to be important for the regulation of systemic dynamics such as immune function [38]. In addition, it is widely accepted that pain signals are transmitted to the brain, and persistent pain may cause dysfunction of the central nervous system. Therefore, the activity in brain regions related to immune regulation could be affected under a state of persistent pain. Although this notion needs to be further verified by future studies, such central and peripheral modulations may orchestrally contribute to the promotion of tumor growth by persistent pain.

In conclusion, we demonstrated here that persistent pain signals promoted tumor growth in mice. Furthermore, activated sensory neurons in tumor-bearing mice may induce the increased expression of pain-related peptides, such as SP, CGRP, and VEGF in sensory neurons, which could promote tumor growth. Moreover, we revealed that the inhibition of pain transmission was sufficient to improve the survival period under severe cancer pain. These findings provide evidence that controlling the activity of sensory neurons at the early stage of cancer is a key strategy for cancer patients to suppress tumor progression.

## Supplementary Information

**Additional file 1: Table S1.** Details of RT-qPCR primer.

## Data Availability

All of the data generated and analyzed in this study are included in this published article.

## Abbreviations

AAV: Adeno-associated virus
AXT: Accelerated bone formation cell-derived severe osteosarcoma
B16: B16 melanoma
Calca: Calcitonin-related polypeptide alpha
CGRP: Calcitonin gene-related peptide
CNO: Clozapine *N*-oxide
DREADD: Designer receptors exclusively activated by designer drugs
DRG: Dorsal root ganglion
ERKs: Extracellular signal-regulated kinases
GAPDH: Glyceraldehyde 3-phosphate dehydrogenase
hM3Dq: Mutated Gq-coupled human M3 muscarinic receptor
hSyn: Human synapsin 1
IVIS: In vivo imaging system
LLC: Lewis lung carcinoma
NGS: Normal goat serum
QOL: Quality of life
RT-qPCR: Quantitative reverse transcription polymerase chain reaction
SP: Substance P
STAT3: Signal transducer and activator of transcription 3
Tac1: Tachykinin precursor 1
TME: Tumor microenvironment
Vegfa: Vascular endothelial growth factor a

## References

[1] Virgen CG, Kelkar N, Tran A, Rosa CM, Cruz-Topete D, Amatya S (2022). Pharmacological management of cancer pain: novel therapeutics. Biomed Pharmacother.

[2] Garzón-Rodríguez C, Lyras L, Gayoso LO, Sepúlveda JM, Samantas E, Pelzer U (2013). Cancer-related neuropathic pain in out-patient oncology clinics: a European survey. BMC Palliat Care.

[3] Weilbaecher KN, Guise TA, McCauley LK (2011). Cancer to bone: a fatal attraction. Nat Rev Cancer.

[4] Andriessen AS, Donnelly CR, Ji RR (2021). Reciprocal interactions between osteoclasts and nociceptive sensory neurons in bone cancer pain. Pain Rep.

[5] van den Beuken-van Everdingen MH, Hochstenbach LM, Joosten EA, Tjan-Heijnen VCG, Janssen DJA (2016). Update on prevalence of pain in patients with cancer: systematic review and meta-analysis. J Pain Symptom Manage.

[6] Chwistek M (2017). Recent advances in understanding and managing cancer pain. F1000Res..

[7] Hadi MA, McHugh GA, Closs SJ (2019). Impact of chronic pain on patients' quality of life: a comparative mixed-methods study. J Patient Exp.

[8] Filipponi C, Masiero M, Pizzoli SFM, Grasso R, Ferrucci R, Pravettoni G (2022). A comprehensive analysis of the cancer chronic pain experience: a narrative review. Cancer Manag Res.

[9] Coleman RE (2006). Clinical features of metastatic bone disease and risk of skeletal morbidity. Clin Cancer Res.

[10] Torrance N, Elliott AM, Lee AJ, Smith BH (2010). Severe chronic pain is associated with increased 10 year mortality. A cohort record linkage study. Eur J Pain..

[11] Deplanque G, Demarchi M, Hebbar M, Flynn P, Melichar B, Atkins J (2015). A randomized, placebo-controlled phase III trial of masitinib plus gemcitabine in the treatment of advanced pancreatic cancer. Ann Oncol.

[12] Temel JS, Greer JA, Muzikansky A, Gallagher ER, Admane S, Jackson VA (2010). Early palliative care for patients with metastatic non-small-cell lung cancer. N Engl J Med.

[13] Faulkner S, Jobling P, March B, Jiang CC, Hondermarck H (2019). Tumor neurobiology and the war of nerves in cancer. Cancer Discov.

[14] Gysler SM, Drapkin R (2021). Tumor innervation: peripheral nerves take control of the tumor microenvironment. J Clin Invest.

[15] Zahalka AH, Frenette PS (2020). Nerves in cancer. Nat Rev Cancer.

[16] Watanabe M, Narita M, Hamada Y, Yamashita A, Tamura H, Ikegami D (2018). Activation of ventral tegmental area dopaminergic neurons reverses pathological allodynia resulting from nerve injury or bone cancer. Mol Pain.

[17] Sato D, Narita M, Hamada Y, Mori T, Tanaka K, Tamura H (2022). Relief of neuropathic pain by cell-specific manipulation of nucleus accumbens dopamine D1- and D2-receptor-expressing neurons. Mol Brain.

[18] Katsuda Y, Tanaka K, Mori T, Narita M, Takeshima H, Kondo T (2021). Histone modification of pain-related gene expression in spinal cord neurons under a persistent postsurgical pain-like state by electrocautery. Mol Brain.

[19] Kondo T, Hamada Y, Sato D, Tanaka K, Yamabe Y, Narita M (2020). Conditional activation of peripheral sensory nerves induces an aversive state with the down-regulation of neural functions of the nucleus accumbens. Jpn J Pharm Palliat Care Sci.

[20] Imai S, Ikegami D, Yamashita A, Shimizu T, Narita M, Niikura K (2013). Epigenetic transcriptional activation of monocyte chemotactic protein 3 contributes to long-lasting neuropathic pain. Brain.

[21] Shimizu T, Ishikawa T, Sugihara E, Kuninaka S, Miyamoto T, Mabuchi Y (2010). c-MYC overexpression with loss of Ink4a/Arf transforms bone marrow stromal cells into osteosarcoma accompanied by loss of adipogenesis. Oncogene.

[22] Shimizu T, Sugihara E, Yamaguchi-Iwai S, Tamaki S, Koyama Y, Kamel W (2014). IGF2 preserves osteosarcoma cell survival by creating an autophagic state of dormancy that protects cells against chemotherapeutic stress. Cancer Res.

[23] Sato D, Hamada Y, Narita M, Mori T, Tezuka H, Suda Y (2022). Tumor suppression and improvement in immune systems by specific activation of dopamine D1-receptor-expressing neurons in the nucleus accumbens. Mol Brain.

[24] Gondoh E, Hamada Y, Mori T, Iwazawa Y, Shinohara A, Narita M (2022). Possible mechanism for improving the endogenous immune system through the blockade of peripheral μ-opioid receptors by treatment with naldemedine. Br J Cancer.

[25] Chen KH, Yang CH, Cheng JT, Wu CH, Sy WD, Lin CR (2010). Altered neuronatin expression in the rat dorsal root ganglion after sciatic nerve transection. J Biomed Sci.

[26] Iyer SM, Vesuna S, Ramakrishnan C, Huynh K, Young S, Berndt A (2016). Optogenetic and chemogenetic strategies for sustained inhibition of pain. Sci Rep.

[27] Mulvey MR, Boland EG, Bouhassira D, Freynhagen R, Hardy J, Hjermstad MJ (2017). Neuropathic pain in cancer: systematic review, performance of screening tools and analysis of symptom profiles. Br J Anaesth.

[28] Andersen KG, Duriaud HM, Jensen HE, Kroman N, Kehlet H (2015). Predictive factors for the development of persistent pain after breast cancer surgery. Pain.

[29] Wang L, Cohen JC, Devasenapathy N, Hong BY, Kheyson S, Lu D (2020). Prevalence and intensity of persistent post-surgical pain following breast cancer surgery: a systematic review and meta-analysis of observational studies. Br J Anaesth.

[30] Chang WK, Tai YH, Lin SP, Wu HL, Tsou MY, Chang KY (2019). An investigation of the relationships between postoperative pain trajectories and outcomes after surgery for colorectal cancer. J Chin Med Assoc.

[31] Carmeliet P (2005). VEGF as a key mediator of angiogenesis in cancer. Oncology.

[32] Goel HL, Mercurio AM (2013). VEGF targets the tumour cell. Nat Rev Cancer.

[33] Elaimy AL, Mercurio AM (2018). Convergence of VEGF and YAP/TAZ signaling: implications for angiogenesis and cancer biology. Sci Signal..

[34] Gutierrez S, Boada MD (2018). Neuropeptide-induced modulation of carcinogenesis in a metastatic breast cancer cell line (MDA-MB-231LUC+). Cancer Cell Int..

[35] Deng XT, Tang SM, Wu PY, Li QP, Ge XX, Xu BM (2019). SP/NK-1R promotes gallbladder cancer cell proliferation and migration. J Cell Mol Med.

[36] Zhang Y, Lin C, Liu Z, Sun Y, Chen M, Guo Y (2022). Cancer cells co-opt nociceptive nerves to thrive in nutrient-poor environments and upon nutrient-starvation therapies. Cell Metab.

[37] Zhu W, Sheng D, Shao Y, Zhang Q, Peng Y (2021). Neuronal calcitonin gene-related peptide promotes prostate tumor growth in the bone microenvironment. Peptides.

[38] Pavlov VA, Tracey KJ (2017). Neural regulation of immunity: molecular mechanisms and clinical translation. Nat Neurosci.

