# Supplementary material for: Cancer aggravation due to persistent pain signals with the increased expression of pain-related mediators in sensory neurons of tumor-bearing mice
Source: Mol Brain. 2023 Feb 3;16:19. doi: 10.1186/s13041-023-01001-5 (PMC9896755; doi:10.1186/s13041-023-01001-5)
Supplement: Supplementary file 1 — Additional file 1: Table S1. Details of RT-qPCR primer. [file 13041_2023_1001_MOESM1_ESM.pdf]

Table S1. Details of RT-qPCR primer

Gene symbol	Forward primer (5'→3')	Reverse primer (5'→3')
Bdnf	CGAGAGGTCTGACGACGACAT	TCCGCGTCCTTATGGTTTTC
Calca	TCCTGAAGTTCTCCCCTTTCC	GGGCTGCTTTCCAAGATTGA
Gapdh	CATGGCCTTCCGTGTTCCTA	GATGCCTGCTTCACCACCTT
Gdnf	GGATGGGATTCGGGCCACT	AGCCACGACATCCCATAACTTC
Ngf	GACCACAGCCACAGACATCAA	GCTCGGCACTTGGTCTCAAA
Tac1	AATCGATGCCAACGATGATCT	GGGCGATTCTCTGCAGAAGA
Vegfa	GAAGCTACTGCCGTCCGATT	GATCCGCATGATCTGCATGG
